# Esophageal Perforation due to Anterior Cervical Spine Hardware Placement: Case Series

**DOI:** 10.1155/2019/7682654

**Published:** 2019-06-25

**Authors:** Thomas S. Lee, Eric N. Appelbaum, Derek Sheen, Reintine Han, Benjamin Wie

**Affiliations:** ^1^Virginia Commonwealth University, Department of Otolaryngology-Head and Neck Surgery, USA; ^2^Baylor College of Medicine, Department of Otolaryngology-Head and Neck Surgery, USA; ^3^Hackensack Meridian Health Mountainside Medical Center, Department of Family Medicine, USA; ^4^Wake Forest University School of Medicine, USA

## Abstract

*Context. *This case series discusses surgical management of esophageal perforations that occurred following cervical spine hardware placement.* Purpose. *(1) Determine presenting symptoms of esophageal perforation after anterior cervical spine hardware placement. (2) Discuss surgical management of these resulting esophageal perforation complications.* Design/Setting. *Case series of six patients at a tertiary-care, academic medical center.* Patient Sample. *Six patients with pharyngoesophageal perforations following anterior cervical spine surgery (ACSS)*. Outcome Measures. *Date of ACSS, indication for ACSS, level of hardware, location of esophageal or pharyngeal injury, symptoms at presentation, surgical intervention, type of reconstruction flap, wound culture flora, and antibiotic choice.* Methods. *A retrospective review of patients with an esophageal or hypopharyngeal injury in the setting of prior ACSS managed by the otolaryngology service at a tertiary, academic center between January 2015 and January 2019.* Results.* Six patients who experienced pharyngoesophageal perforation following ACSS are included in this study. Range of presentation was two weeks to eight years following initial hardware placement. Five patients presented with an abscess and all had evidence of perforation on initial CT or esophagram. All patients underwent repair with a sternocleidomastoid flap with two patients eventually requiring an additional pectoralis myofascial flap for a persistent esophageal leak. Five patients eventually attained ability to tolerate oral nutrition. An algorithm detailing surgical reconstructive management is proposed.* Conclusions. *Esophageal perforations in the setting of prior ACSS are challenging clinical problems faced by otolaryngologists. Consideration should be given to early drainage of abscesses and spine surgery evaluation. Spinal hardware removal is recommended whenever possible. Utilization of a pedicled muscle flap reinforces primary closure and allows coverage of the vertebral bony defect. Nutrition, thyroid repletion, and culture-directed IV antibiotics are necessary to optimize esophageal perforation repair.

## 1. Introduction

An anterior approach to the cervical spine with placement of spinal hardware is one of the most commonly performed spine surgeries. Pharyngoesophageal perforation related to anterior cervical spine surgery (ACSS) is rare with unknown prevalence but can have significant morbidity and mortality (3.92%) [1]. Delayed presentation of a perforation has been reported in 0.2 to 1.5% of cases [1, 2]. Average time to diagnosis is 2 years (0 days to 18 years) [1, 2].

Delayed perforations may be attributed to several causes, but the most important etiology is the migration or fracture of hardware (41%), followed by chronic erosion via hardware mass effect (31%) [1–4]. Chronic pressure from hardware can cause tissue ischemia or formation of diverticulum, thus weakening the posterior esophageal wall [1–4]. Other etiologies of perforation include intraoperative injury (19%) and graft extrusion and penetration (7%) [1].

Common presenting symptoms of acute esophageal perforations include pain, dysphagia, aspiration, and hoarseness. Delayed esophageal erosions require a high level of suspicion as presenting signs and symptoms may be nonspecific. Imaging studies can be helpful in diagnosis but an esophageal injury was identified in only 72.7% of patients in reported studies [5]. The current study represents a case series of six patients with pharyngoesophageal perforation following ACSS.

## 2. Materials and Methods

This study was a retrospective review of patients with an esophageal or hypopharyngeal injury in the setting of prior ACSS managed by the otolaryngology service at a tertiary, academic center between January 2015 and January 2019. Charts were reviewed for relevant information including date of ACSS, indication for ACSS, level of hardware, location of esophageal or pharyngeal injury, symptoms at presentation, surgical intervention, type of reconstruction flap used, wound culture flora, and antibiotic choice. Date of intervention was defined as the date of primary pharyngoesophageal repair with or without muscle flap. Resolution was defined as achievement of oral intake after an esophagram demonstrated absence of a leak. This study was approved by and performed in accordance with Institutional Review Board guidelines.

## 3. Results

A total of 6 patients who experienced pharyngoesophageal perforation were treated by the otolaryngology service from January 2015 to January 2019, including 3 females and 3 males with ages ranging from 26 to 80 years old (Table 1). Indications for the initial spine surgery include cervical spine trauma, cervical spine degeneration, and malignancy. The average time to presentation with an esophageal perforation following the patient's most recent cervical spine procedure ranged from 12 days to 8.7 years (mean: 2.2 years). Upon presentation, common signs and symptoms of esophageal perforation included dysphagia, neck pain, and radiographic findings suggestive of perforation in five out of six patients (Patients #2-6) (Table 2). The remaining patient (Patient #1) presented with a dislodged screw in her right mainstem bronchus, underwent repair for the tracheal defect, and later developed a subsequent esophageal defect due to erosion into a deep vertebral body defect.

Intraoperatively, all patients underwent joint exploration with spine surgery for possible hardware removal. In all cases, the location of the esophageal perforation occurred at the site of the hardware. Three patients (Patients #2, #3, and #6) were found to have diverticula intimately associated with the cervical hardware and surrounding bony defect.

Four of six patients required multiple surgeries for their esophageal perforation repair. Five of six patients underwent hardware removal at the time of the first reconstructive surgery addressing the esophageal perforation. Of the two patients who did not have their hardware removed initially, one (Patient #2) eventually required removal during a subsequent intervention due to persistent infection and poor wound healing. Due to his recent hardware placement two weeks prior to presentation, Patient #5 was able to keep his hardware in place and attained oral intake. All other cases presented in a delayed fashion more than 30 days after the initial ACSS. Notably, one patient (Patient #4) had spine hardware replaced with new hardware at the time of the initial esophageal repair surgery but eventually required total hardware removal due to persistent infection and esophageal perforation.

**Figure 1:**
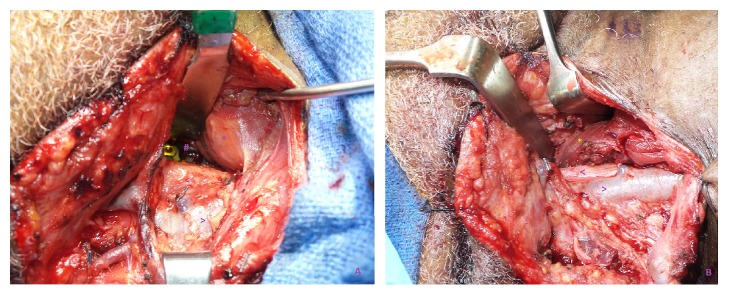
(a) Right neck with spine hardware (#) exposed with pharynx and esophagus (∧) being retracted medially. Common carotid artery (<) and internal jugular vein (>) are seen. (b) Inferiorly based SCM flap (*∗*) covering the spine hardware and reinforcing the pharynx and esophageal repair.

Regarding reconstruction, all patients eventually underwent placement of a regional muscular flap for reinforcement of the esophageal perforation repair. An SCM flap was the most common choice, while a pectoralis flap was used in cases where the initial SCM flap had failed, a large vertebral body defect at the site of hardware removal was present, or the esophageal perforation was too large (>3x3cm, or inability to reapproximate mucosal flaps without excessive tension) (Figure 1). Patients #1 and #4 eventually required use of a pectoralis major rotational flap for reconstruction of persistent pharyngoesophageal defects despite initial SCM flap placement. All patients underwent culture-directed IV antibiotic therapy in consultation with the infectious disease service. Wound cultures taken from abscess and perforation sites were variable (Table 3).

All patients were kept on an NPO diet until an esophagram demonstrated resolution of esophageal perforation. Enteral nutrition in the form of high-calorie, high-protein tube feeding diet was initiated via nasogastric or gastrostomy tube placement. Once the absence of an esophageal leak was confirmed on esophagram with contrast, patients were started on a graduated oral diet. Time to oral intake from the latest procedure ranged from 7 to 62 days (mean: 22 days). Patient #3 did not attain oral intake prior to his death from a perioperative myocardial infarction.

## 4. Discussion

Pharyngoesophageal perforations present complex surgical challenges and represent a feared complication of ACSS. The sternocleidomastoid (SCM) flap may be used to reinforce primary closure of the esophageal perforation and has proved to be effective as a definitive treatment option for esophageal fistula after ACSS [6–8]. According to Halani et al.'s systemic review of literature from 1980 through 2015, the SCM flap reinforcement was the most commonly used method (n=35), followed by the pectoralis flap (n=4), radial forearm free flap (n=4), omental flap (n=3), infrahyoid muscle (n=2), omohyoid muscle (n=2), latissimus dorsi (n=1), longus colli (n=1), and jejunum (n=1). Primary closure was performed in 31 patients and conservative treatment without surgery was done in 12 patients [1]. Time to achieve oral intake was comparable between primary closure (28.3 days) and SCM flap (27.3 days), while conservative management was associated with a significant delay (68 days) [1]. This case series adds to the body of literature supporting the use of a SCM or a pectoralis flap to repair and reinforce infected esophageal perforations in the setting of ACSS hardware presence [1–3]. The reason for supporting use of a flap is that it provides a physical barrier and increased arterial supply, offering increased healing and delivery of antibiotics to the wound site [9].

**Figure 2:**
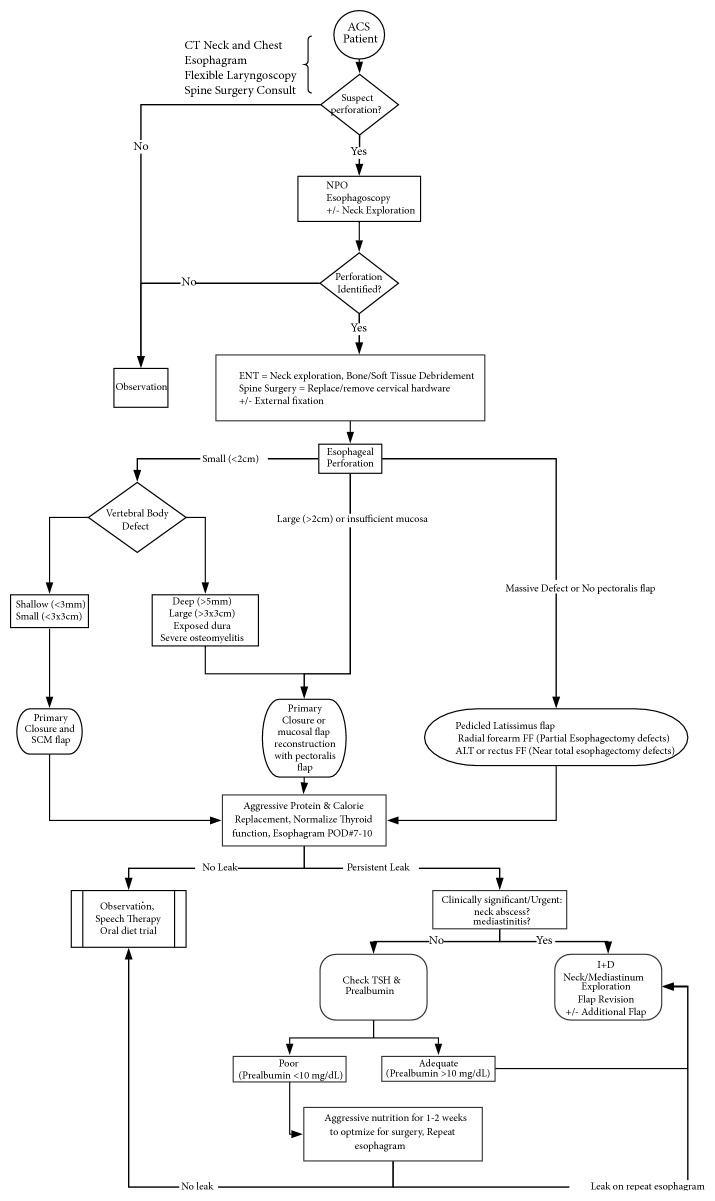
Esophageal perforation management algorithm. ACSS = anterior cervical spine surgery. CT = computed tomogram. NPO =* nil per os*. SCM = sternocleidomastoid. POD = postoperative day. FF = free flap. I+D = incision and drainage of abscess. This algorithm provides a general guideline for flap selection. However, because the donor flap bulk varies between patients, care must be taken to individualize flap choice.

Managing this relatively rare but devastating complication can present a unique surgical challenge for a reconstructive surgeon. A general treatment algorithm utilized by the senior author is proposed (Figure 2), which is based on retrospective experience. A reconstructive surgeon should individualize each patient's treatment based on multiple factors, including nutritional status, thyroid function, timing from ACSS, and available reconstructive options. Spine surgery, infectious disease, and nutrition consultations should be considered. In ACSS patients, one should maintain a high level of suspicion of a pharyngoesophageal perforation with symptoms of neck pain, dysphagia, dyspnea, hoarseness, or abscess [5].

Based on patient complaints, one may consider a diagnostic work-up that includes CT neck and chest, esophagram, and flexible laryngoscopy. There is a differing opinion as far as the best diagnostic workup between the contrast esophagram versus CT/MRI neck as a primary diagnostic modality to rule out esophageal perforations [9–13]. Perrone et al. cite the contrast CT and MRI of the neck to be the gold standard with 92-100% sensitivity to investigate for the neck, mediastinum, spine, and prostheses. However, it is important to consider a controversy regarding varying degrees of false negativity (up to 25%) for the contrast esophagram [10]. In consensus with other authors, we do not recommend the use of flexible esophagoscopy with air insufflation given risk of enlarging perforations. [9]. Our recommendation is that there should be a combination of studies to obtain the maximum amount of information before deciding to perform a neck exploration and we performed a CT neck with contrast as well as a contrast esophagram prior to surgical exploration. In our case series, most patients presented with an abscess at the site of the hardware and all patients were found to have a pharyngoesophageal perforation directly overlying cervical hardware based on intraoperative findings. Therefore, we recommend a low threshold to consider esophagoscopy and neck exploration in coordination with a spine surgeon. Hershman et al. additionally recommend that if an esophageal perforation is suspected clinically, the next best step is to surgically explore with early and aggressive management. It is important to note that simple incision and drainage without attempted esophageal repair will likely fail [13, 14]. Several authors also recommend complete spinal hardware removal in delayed perforations, diagnosed at greater than 1 month from original anterior cervical surgery [12]. Additionally, if there is evidence of a lack of vertebral fusion, Harman et al. recommended posterior fusion along with a repair of the esophageal perforation with a flap. In our Patient #1, we removed hardware completely and performed a temporary halo external fixator to stabilize the spine and subsequently performed a staged posterior fusion in a secondary surgery. Perrone et al. likewise recommend complete removal of hardware in the setting of extensive infection. These authors also recommend using a posterior external or internal fixation procedure if there is no evidence of vertebral fusion. They provide the added suggestion in the setting of lack of vertebral fusion, spine instability, or inefficacy of posterior fixation to perform an anterior spine fixation with either an autologous bone implant or a titanium cage status after bone debridement [10]. Our feeling is that an anterior approach may cause a secondary hardware exposure; therefore caution should be taken when repeating another anterior procedure. Once perforation is confirmed, open neck exploration should be considered. There is a controversy with when to employ conservative careful observation vs early surgical intervention [13, 14]. It appears that some authors recommend conservative management when the perforation is small (defined as <1cm in diameter) and surgical intervention when the perforation is large (>1cm) [10, 12]. Perrone et al. cited a 12% mortality rate with surgery compared with an 18% mortality rate with conservative management. In general, the bottom line of early and aggressive management for everyone is recommended by most authors, given the mortality rate of treatment <24 hours being 20% versus that of treatment >24 hours being close to 50% [13]. Therefore, our feeling is to generally lean towards surgical intervention especially when deciding to manage conservatively, with the esophageal perforation failing to resolve itself within one week of observation. The threshold for surgical intervention however is still a controversy [10, 12]. Given the difficulty to assess the exact size of the esophageal perforation until at least a rigid esophagoscopy is performed in the operating room, there is uncertainty with categorizing a perforation to be <1cm or >1cm. In our case series at the time of identification of a perforation using rigid esophagoscopy, we immediately performed a neck exploration with a flap repair. It is important that the approach for neck exploration should be performed on the same side as the previous ACSS. Flexible laryngoscopy performed prior to neck exploration will therefore be helpful because a neck exploration should always be performed on the side of vocal cord dysfunction to protect the intact contralateral recurrent laryngeal nerve [15].

The majority of the patients in this case series presented in a delayed fashion, averaging 2 years after initial ACSS. The timing of presentation is a crucial factor when considering reconstruction. Early perforations, occurring within 30 days of initial surgery, are generally sustained intraoperatively during placement of cervical hardware [5, 12]. Instrumentation, retraction, or hardware placement may violate the esophageal wall and cause full-thickness injury. If this occurs, intraoperative repair or early recognition of the defect in the postoperative period and conservative management may lead to resolution [16]. In cases of indolent pharyngoesophageal perforation, cervical hardware is thought to cause perforation from chronic tissue ischemia at point of contact with the esophagus [17].

The timing may play an important role in two ways in chronic perforations. Firstly, due to prolonged duration of hardware exposure to saliva and associated pathogens, there is an increased potential for biofilm growth on the hardware which can then be a source for ongoing infection despite aggressive IV microbial therapy and surgical debridement [18]. As such, whenever possible, we advocate for removal of the old spine hardware and avoidance of hardware replacement if possible, which is in agreement with other authors [10, 12]. It is important to note that complete hardware removal or replacement may not always be possible. Cultures from delayed perforations from this case series generally demonstrated multiple species of bacteria and fungus (Table 3). Aggressive, culture-directed IV antimicrobial therapy should be initiated in conjunction with infectious disease consultation.

Secondly, in delayed perforations, adequate bone fusion may allow hardware removal as determined by a spinal surgeon. In early perforations, sufficient bony fusion likely has not occurred and hardware removal may result in unacceptable cervical instability. In situations when hardware removal is absolutely necessary, placement of external fixation and posterior cervical fusion may be considered under the guidance of a spinal surgeon. This is in agreement with the treatment algorithms from both Harman et al. and Perrone et al.

In the setting of poor nutritional status and a grossly infected surgical wound bed, simple primary closure will likely lead to a persistent leak. We advocate placement of a myofascial regional flap overlying the perforation repair site and the vertebral body to optimize healing and to minimize saliva exposure to the vertebral body and dura [1]. Prior to perforation repair, necrotic devascularized tissue should be debrided aggressively until bleeding tissue is encountered. Esophageal mucosal edges should be inverted, as in laryngectomy closures, with multilayer, water-tight technique. Vertebral body defect edges at the site of hardware removal should be debrided, and sharp bony edges should be drilled until smooth in order to minimize the risk of a second or recurrent esophageal perforation, as seen in our Patient #1.

In our treatment algorithm, the selection of regional flaps depends on the size of the esophageal perforation, vertebral body defect, and the size of the donor flap. The SCM flap is the most commonly used flap for reconstruction of esophageal perforation [1, 9–12]. In this case series, the SCM flap was also most frequently used as the initial flap used for reconstruction. An SCM flap is ideal for smaller defects and has an advantage of occupying the same surgical field with relatively minor morbidity [19]. Both inferiorly- or superiorly-based flaps have been performed and appear to have similar efficacy (though the inferiorly base blood supply is more consistent) [20]. The decision on flap blood supply (inferior vs superior) should be based on the flap reach. Barlow et al. described use of this flap to reconstruct defects up to *½*  the circumference of the patient's esophagus [21]. Advantages include tissue pliability, minimal excess bulk reducing cosmetic deformities, and low donor site morbidity.

We recommend using bulkier tissue flaps such as the pectoralis flap in the setting of esophageal perforations >2cm, vertebral body defects greater than 5mm, or SCM flaps that have failed to resolve the esophageal perforation [1, 11]. For a large esophageal perforation (>2cm) or in situation where the vertebral body defect is large (3x3cm) or deep/protruding (>5mm), we recommend placing a bulky myofascial flap to sufficiently reinforce the esophageal perforation repair site while obliterating the vertebral body defect. Based on our experience, the pectoralis major myofascial (PMMF) flap provides the necessary tissue bulk for these situations. A deepithelialized musculocutaneous pectoralis flap can also be utilized to obtain additional tissue bulk derived from subdermal tissue. In our series, two patients (Patients #1, 4) required pectoralis major flaps following failed SCM flap reconstruction. In both cases, repair of the perforation was complicated by sepsis, chronic surgical site infection, and multiple medical comorbidities. Despite the increased bulk of the PMMF, both patients were able to attain meaningful oral nutritional intake.

Although the treatment algorithm provides a general outline, ultimately the size of the donor flap will determine the individualized reconstruction. In some individuals with a muscular neck, the SCM flap alone may be sufficient if the SCM flap has sufficient muscle bulk to obliterate a deep bony defect extending towards the dura. Contrastingly, in small, thin patients without sufficient muscle bulk, a typical defect that can be managed with an SCM flap may require a pectoralis flap to recruit additional tissue bulk. If the pectoralis flap is very thin and needs additional bulk, it can be raised with the skin, underlying subcutaneous fat and muscle. The skin can be incorporated as part of the mucosal reconstruction (if there is a sizable full thickness mucosal defect) or deepithelialized and buried deep to the mucosal closure. The muscle can be used to reinforce the esophageal perforation repair site or to obliterate the vertebral body defect. In patients with massive pectoralis muscle bulk, one can choose to raise without skin or subdermal tissue to minimize excess bulk. Narrowing down the muscle cuff around the pedicle will optimize rotation of the pedicle, yet it will minimize the amount of flap bulk being harvested.

If a pectoralis flap is not available, one may consider using a pedicled latissimus flap, which can easily reach the esophageal perforation site [1, 11, 22]. These may be designed overlying the latissimus muscle close to the hip approaching midline to ensure sufficient flap reach. Once the thoracodorsal artery is identified, the serratus branch and circumflex scapular artery can be sacrificed to ensure no limitation on rotation of the flap. The senior author (T.L.) releases the lateral border of the pectoralis major muscle (if present) while preserving the thoracoacromial artery blood supply to the pectoralis major muscle. This prevents the latissimus flap pedicle from being compressed as the latissimus flap gets tunneled subcutaneously toward the neck.

Generally, regional pedicled flaps are preferred as they have more reliable blood supply than free flap reconstruction. However, in the absence of an available pedicled flap or if a much larger esophageal defect is present, free flap (FF) transfer has been described for use in reconstruction [1, 11, 23, 24]. A radial forearm FF is ideal for full thickness esophageal wall defects (typically 6-7 cm by 9-12 cm) while even larger defects consisting of near total esophageal defect as commonly seen after total pharyngectomy/total laryngectomy would require an anterolateral thigh (ALT) or rectus FF to reconstruct the entire cervical esophagus from pharynx or tongue base down to the base of the neck. Free flap transfer must be approached with caution in this patient population. Adequate neck donor veins may be severely limited due to chronic infection predisposing to venous thrombosis of large vessels including the internal jugular vein. One may consider utilizing the contralateral neck for vessels but this may compromise the safety of the uninvolved recurrent laryngeal nerve. Even after a successful venous anastomosis, the reconstructive surgeon should monitor closely for thrombotic compromise of the flap vessels due to prolonged infection of the wound bed [25].

Lastly, culture-directed antimicrobial therapy and aggressive nutritional and thyroid repletion are paramount to successful healing [9, 10, 14]. Broad-spectrum IV antibiotic and antifungal treatments are among the initial therapies, with intraoperative tissue cultures further directing targeted therapy. Kang et al. recommended using broad spectrum antibiotics to cover for multiple organisms including gram positive, gram negative, and anaerobic organisms secondary to gastric content contamination. Regarding the duration of antibiotics, there have been no specific durations of time recommended with some authors suggesting use of 3 weeks for poor surgical candidates [11]. Other authors have suggested extending broad spectrum antibiotic usage for up to one year for patients with evidence of osteomyelitis [12]. Perrone et al. recommended IV antibiotics until white blood cell count and C-reactive protein have normalized on lab values. In general, our recommendation is that consultation with infectious disease specialists is advised. Postoperatively, antibiotic therapy should be tailored based on intraoperative tissue cultures.* Staphylococcus aureus, Candida, Pseudomonas*, and* Streptococcus* species have been isolated in this case series, which aligns with a systematic review conducted by Halani* et al. *(Table 3) [1].

The importance of nutrition and thyroid dysfunction cannot be stressed enough. TSH is checked weekly with aggressive thyroid replacement using IV or PO levothyroxine. For nutrition replenishment, enteric feeds are preferable to parenteric feeds [26]. For patients who are profoundly malnourished, placement of a gastric feeding tube is favored over nasogastric tube to optimize nutrition and to minimize pressure applied to the esophageal repair site. Malnutrition and low prealbumin have been found to be a risk factor for poor healing outcomes in head and neck reconstruction procedures [27]. We routinely check prealbumin and nitrogen balance in consultation with an experienced nutritionist to ensure continued anabolic state. In the absence of a clinically urgent problem such as mediastinitis, malnutrition may cause deferment of revision surgery until the nutritional status improves (Figure 2). As commonly seen in head and neck oncologic reconstruction, poor nutritional status often leads to a salivary fistula and must be addressed when attempting to maximize healing potential [28]. Clinical judgment should be employed when assessing nutritional and thyroid function status and determining when to consider revision surgery as many patients may not attain normal nutritional state. One must also be familiar with the limitation of prealbumin in assessing adequate nutrition in the setting of ongoing inflammation. In our patients, improved nutritional status and aggressively treated hypothyroidism often correlated with resolution of an esophageal leak. Overall, enteric replenishment of nutrition is preferable to parenteric if possible. Immediately following the first repair for the first 1-2 weeks, aggressive protein and caloric replenishment should begin regardless of prealbumin levels, while the patient remains NPO pending a repeat esophagram. In 1-2 weeks, a repeat esophagram is obtained to evaluate for any persistent leaks. During this time, emergent settings including severe mediastinitis or neck abscess would require an immediate operation regardless of nutritional status. However, in nonemergent settings we would further optimize thyroid function and check prealbumin levels. With prealbumin levels <10 mg/dL, surgery is deferred to further optimize nutrition; with levels >10mg/dL then it is possible to consider revision surgery without further delays. Aggressive protein replacement (1.7-2 gram/kg/day) and calories are provided to create anabolic state to help optimize wound healing. Aggressive nutrition should be considered to optimize wound healing even if prealbumin is not low but this is especially important if the prealbumin is low. NPO status is essential until resolution of perforation is confirmed on esophagram. Therefore in summary, premature diet initiation can weaken the repair site and may lead to recurrence of perforations.

## 5. Conclusion

Our study represents a case series of pharyngoesophageal perforations in the setting of anterior cervical spine surgery. Patients with a history of ACSS presenting with complaints of dysphagia, neck pain, neck swelling, or signs of infection should be evaluated with a suspicion for pharyngoesophageal perforation and be referred for radiologic studies prior to surgery. Patients may present in an early or delayed fashion. Close coordination with a spine surgeon is recommended to evaluate for removal of cervical hardware, if clinically appropriate. We advocate for the utilization of regional myofascial flaps to assist in perforation closure. The SCM flap is an attractive option for reconstruction due to its location, pliability, and low donor site morbidity. Some perforations may require use of pectoralis flap if there is a large esophageal perforation, large vertebral body defect, or persistent leak after a prior reconstruction. A multispecialty approach is essential in managing these complex surgical patients.

## Figures and Tables

**Table 1:** Summary of patient data. Patient #5 had fixation at multiple levels. ACS = anterior cervical spine. MVC = motor vehicle collision. Time to presentation refers to interval since most recent ACS surgery. PR = primary defect repair. I&D = incision and drainage of abscess. HR = hardware removal. SCM = sternocleidomastoid rotational flap. rSCM = revision SCM flap; PMMF = pectoralis major myofascial flap. Time to oral intake refers to interval from most recent reconstructive surgery intervention to resumption of oral intake.

Patient	Age	Gender	Levels of Fixation	Reason for ACS surgery	Time to Presentation	Intervention	Time to Oral Intake
1	34	F	C4-7	MVC, compression fracture, chronic epidural abscess	52 days	1. PR + HR + SCM 2. PR + PMMF	17 days

2	58	F	C5-6	cervical spondylosis	67 days	1. I&D + PR + SCM 2.I&D + PR + HR +rSCM	7 days

3	80	M	C4-5	Cervical degeneration, odontoid fracture	3.35 years	1. PR + HR + SCM	Did not attain

4	26	F	C5-6, T1-3	MVC	1.29 years	1. I&D + HR 2. I&D 3. I&D + PR + HR + SCM 4. I&D + PR + PMMF	62 days

5	56	M	C2-3, C4-5, C6-7	cervical spine degeneration	12 days	1. I&D + PR + SCM	10 days

6	70	M	C5-6	cervical spine degeneration	8.70 years	1. PR + HR + SCM	8 days

**Table 2:** Patient symptoms at presentation.

Symptom	Number of patients
Dyspnea	1

Fever	1

Recurrent pneumonia	1

Regurgitation	1

Sepsis	1

Cough	2

Neck swelling	2

Abscess	5

Cervicalgia	5

Dysphagia	5

Radiographic findings	5

**Table 3:** Summary of patient cultures and antibiotic therapies.

Patient	Culture	Antibiotic
1	MRSA, *Klebsiella*, *E. coli*	ceftaroline, daptomycin, linezolid, rifampin

2	*Klebsiella oxytoca*, *Viridans Streptococcus*	piperacillin-tazobactam, vancomycin

3	*Streptococcus anginosus*, *Klebsiella oxytoca*	levofloxacin, meropenem

4	**Wound:** mixed respiratory flora, *Candida glabrata*, **Respiratory:***Serratia marcescens*, *Pseudomonas aeruginosa*, **Urine:** vancomycin-resistant *Enterococcus*	colistimethate, levofloxacin, meropenem, micafungin, piperacillin-tazobactam, vancomycin

5	Mixed Respiratory Flora	levofloxacin, meropenem, vancomycin

6	Mixed Respiratory Flora	ertapenem, meropenem

## Data Availability

The patient information and data used to support the findings of this study are available from the corresponding author upon request.
